# Effects of Different Water and Nitrogen Supply Modes on Peanut Growth and Water and Nitrogen Use Efficiency under Mulched Drip Irrigation in Xinjiang

**DOI:** 10.3390/plants12193368

**Published:** 2023-09-24

**Authors:** Jianshu Dong, Zhu Xue, Xiaojun Shen, Ruochen Yi, Junwei Chen, Qiang Li, Xianfei Hou, Haocui Miao

**Affiliations:** 1 College of Water Conservancy Engineering, Tianjin Agricultural University, Tianjin 300392, China; dongjianshu_1010@163.com (J.D.); xuezhu@tjau.edu.cn (Z.X.); yiruochen1@163.com (R.Y.); chenjunwei202204@163.com (J.C.); 2 Institute of Economic Crops, Xinjiang Academy of Agricultural Sciences, Urumqi 830091, China; hou544805196@163.com (X.H.); mc09876@163.com (H.M.)

**Keywords:** peanuts, irrigation water quota, water use efficiency, nitrogen use efficiency, drip irrigation

## Abstract

The optimization of irrigation and fertilization indexes for peanuts with drip irrigation is urgently needed in Xinjiang. A field experiment was conducted during the 2021 peanut growing season at Urumqi, Xinjiang, in Northwestern China, to evaluate the effects of different water and nitrogen treatments on the growth, yield, and water and nitrogen utilization of peanuts. In field experiments, we set up three irrigation levels (irrigation water quotas of 22.5, 30, and 37.5 mm, respectively, for W_1_, W_2_, and W_3_), two nitrogen application levels (77.5 and 110 kg·ha^−1^, recorded as N_1_ and N_2_), and a control treatment (W_2_N_0_) that did not include the application of nitrogen. The results showed that nitrogen application enhanced the growth, physiological indexes, yield, and water use efficiency of the W_1_, W_2_, and W_3_ treatments when the irrigation volume remained the same. In comparison with no nitrogen application (W_2_N_0_), the peanut growth, physiological indexes, yield, and water use efficiency improved with increasing irrigation amounts in the N_1_ and N_2_ treatments. With an increase in the irrigation volume, the water use efficiency grew; the W_3_N_2_ treatment had the highest water use efficiency, which was 1.32 kg·m^−3^. The total water consumption and reproductive-stage water consumption of the peanuts in all treatments increased with the irrigation volume, and a high yield was achieved at 402.57 mm, which was 5.2974 Mg·ha^−1^. In the W_1_, W_2_, and W_3_ treatments, the nitrogen partial factor productivity significantly decreased as the nitrogen application increased, with the nitrogen partial factor productivity in the W_3_N_1_ treatment being the highest, at 60.61 kg·kg^−1^. A comprehensive evaluation based on principal component analysis assigned W_3_N_2_ the higher score. These findings suggest that irrigation water quotas of 37.5 mm should be coupled with 110 kg·ha^−1^ nitrogen applications for peanuts using drip irrigation in mulch film in Xinjiang.

## 1. Introduction

In China, peanuts (*Arachis hypogaea* L.) represent an important cash crop that occupies a key position in the country’s economy, and the development of peanut production contributes to reducing insufficient edible oils and fats [1]. In 2021, the national peanut planting area was 4,805,300 hectares, and the total peanut output was 18,307,800 tons. This was with an average yield of 3809.93 kg per hectare, with Xinjiang ranking second in the country with 4886.38 kg per hectare [2]. Xinjiang has abundant light and heat resources, significant temperature differences between day and night, and a dry climate, providing a better natural ecological environment for high-quality and high-yield peanuts. Regarding pests and diseases, *Aspergillus flavus* is the least common example that threatens the quality of peanut products [3]. As a result of its arid climate and limited irrigation resources, Xinjiang is an atypical irrigation area with significant supply–demand contradictions, and the lack of irrigation resources has restricted local agriculture development [4]. An adequate fertilizer dosage can contribute to high yields, promote the accumulation of dry matter and nutrients in crops, and affect the accumulation and translocation of dry matter. Water and fertilizer are indispensable factors in crop growth [5]. Drip irrigation and water–fertilizer integration technology represent one of the most efficient water-saving measures currently available [6]. Drip irrigation–fertilization technology under membrane conditions can provide precision irrigation and fertilization, significantly increasing the efficiency of water and fertilizer utilization and improving crop yields and quality by leveraging the synergistic interaction between them. To achieve sustainable agriculture goals, it is important to optimize the synergistic effects of irrigation and nitrogen fertilization.

Since 1980, the application of nitrogen fertilizer in China has increased dramatically, and the proportion of nitrogen input has reached a level that reduces crop yield returns [7]. Excessive N applications tend to lead to an imbalance in the root–crown ratio of the crop and a significant reduction in indicators such as the yield and nitrogen fertilizer use efficiency [8]. Previous studies have shown that appropriate nitrogen applications can significantly improve photosynthesis, water and nitrogen use efficiency, and crop yields [9]. Peanuts are usually grown in the arid and semi-arid regions of Xinjiang. Despite peanuts being drought-resistant, the water demand during their growth and development stages limits peanut yields in this region. Severe drought stress slows crop growth, reduces dry matter accumulation, leads to leaf stomata closure, increases resistance to CO_2_ diffusion within the mesophyll cell, inactivates enzyme activities, reduces photosynthesis and, ultimately, decreases yields [10,11]. However, a proper water supply not only enhances photosynthesis but also promotes the conversion of “sources” to “sinks” and improves nitrogen fertilizer efficiency [12]. Water and fertilizer have a synergistic effect, and appropriate irrigation and fertilization patterns can improve crop yields [13,14]. It was found that the highest yield and water use efficiency in tomatoes can be obtained through saving 25% water and 25% fertilizer via the regulation effect of drip fertigation [15]. Another study found that the synergistic effect of water and nitrogen positively impacts winter wheat growth, water use efficiency (WUE), and yield [16]. Using water and nitrogen two-factor split-plot experiments, previous studies have shown that water and nitrogen have significant interactions with peanut yield, water consumption, and water and nitrogen use efficiency (WUE and NUE) [17]. Scholars have reached similar conclusions about crops such as maize [18], tomatoes [19], and rice [20]. Therefore, understanding the water–nitrogen coupling relationship is the key to producing high-yield and high-quality peanuts, while maintaining an efficient use of water and fertilizer resources. The research on water and nitrogen regulation regarding peanuts is concentrated in Northeastern and North China. The peanut industry in the Xinjiang region is just getting started, with poor soil and the demand for water and nitrogen fertilizer exceeding the actual production. There are few reports on water and nitrogen regulation in peanuts under drip irrigation in Xinjiang. Further research is needed to elucidate the response mechanisms of peanuts to water and nitrogen regulation. Through field experiments combined with theoretical analysis, the authors of this paper focused on the effects of different water and nitrogen regulation values on the growth, yield, and water and nitrogen use efficiency of peanuts under drip irrigation grown in a membrane in Xinjiang. We describe and quantify their responses to these different factors. Based on a comprehensive evaluation, the optimal water and nitrogen regulation indexes were determined to provide a theoretical basis and technical support for local, high-quality peanut production under drip irrigation under membrane conditions.

## 2. Results

### 2.1. Effects of Water and Nitrogen Regulation on Growth and Physiological Indexes of Peanuts under Mulched Drip Irrigation

Table 1 shows the effects of different water and nitrogen treatments on peanut growth indexes in the experiment (the main stem height, first branch length, branch number, shoot dry mass, and leaf area index (LAI)). The results show that the effect of the irrigation volume (W) on the growth index factor alone at the flowering–pegging, pod-setting, and pod-filling stages was extremely significant (*p* ≤ 0.01). The nitrogen (N) applications had highly significant (*p* ≤ 0.01) one-way effects on the main stem height, first branch length, shoot dry mass, and LAI at the flowering–pegging, pod-setting, and pod-filling stages, and had a one-way effect (*p* ≤ 0.05) on the number of branches at the needle stage of flowering–pegging. The interaction between the two (W × N) had a very significant effect on the main stem height at the pod-setting and pod-filling stages and on the shoot dry mass at the flowering–pegging, pod-setting, and pod-filling stages (*p* ≤ 0.01). It also had a significant effect on the branch number at the pod-filling stage, the first branch length at the pod-setting stage, and the LAI at the flowering–pegging, pod-setting, and pod-filling stages (*p* ≤ 0.05).

The peanut growth indexes under different water and nitrogen treatments showed different growth magnitudes with the advancement in fertility. This rapidly accelerated from the seedling stage to the flowering–pegging stage and then slowed down gradually from the flowering–pegging stage to the pod-filling stage. According to the analysis, the peanut main stem height increased with better irrigation water. Compared with other water treatments, the peanut stem height in the W_3_ treatment (irrigation water quota of 37.5 mm) was substantially higher. Compared with W_1_N_1_ (irrigation water quota of 22.5 mm, nitrogen application rate of 77.5 kg·ha^−1^) and W_2_N_1_ (irrigation water quota of 30 mm, nitrogen application rate of 77.5 kg·ha^−1^), the main stem height in the W_3_N_1_ treatment (irrigation water quota of 37.5 mm, nitrogen application rate of 77.5 kg·ha^−1^) increased by 51.02% and 11.28%, respectively. Compared with W_1_N_2_ (irrigation water quota of 22.5 mm, nitrogen application rate of 110 kg·ha^−1^) and W_2_N_2_ (irrigation water quota of 30 mm, nitrogen application rate of 110 kg·ha^−1^), the W_3_N_2_ treatment (irrigation water quota of 37.5 mm, nitrogen application rate of 11 kg·ha^−1^) increased growth by 26.58% and 3.49%, respectively. Increasing the irrigation volume significantly boosted the main stem height. The peanut main stem height also increased with better N applications. Both the N_1_ (irrigation water quota of 22.5 mm) and N_2_ (irrigation water quota of 30 mm) treatments significantly increased the main stem height compared with the N_0_ treatment (which did not involve the application of nitrogen). With the N_2_ treatment, the main stem was higher. At the W_1_ level, the W_1_N_2_ treatment had a 29.01 percent higher main stem height than W_1_N_1_; at the W_2_ level, the W_2_N_2_ treatment plants grew by 16.27% percent and 34.86% percent compared with the W_2_N_1_ and W_2_N_0_ plants (irrigation water quota of 30 mm, without the application of nitrogen), respectively; and, at the W_3_ level, the W_3_N_2_ treatment plants grew 8.13 percent over the W_3_N_1_ plants. The peanut main stem height increased significantly with high nitrogen treatment. Different water and nitrogen treatments had the same effect on the peanut’s lateral branch length as they did on its main stem height. Water and nitrogen applications increased the number of branches, but the treatments’ differences were insignificant. At the same irrigation level, the surface dry matter weight and leaf area index had the following order: N_2_ > N_1_ > N_0_. The near-surface dry matter weight and leaf area index both showed W_2_ > W_3_ > W_1_ at the N_2_ level and W_3_ > W_2_ > W_1_ at the N_1_ level; this indicates that nitrogen fertilization can promote aboveground biomass growth. Within a specific range of N applications, deficit regulation and excessive irrigation can reduce crop transpiration by inhibiting the redundant growth of aboveground biomass. Within a specific range of N applications, deficit regulation and excessive irrigation can reduce crop transpiration by inhibiting the redundant growth of aboveground biomass. Despite the cumulative effect of the seedling stage on the pod-setting stage and, thus, on leaf growth, the difference in water content in the pod-filling stage was still apparent in the leaf areas of peanuts during the pod-filling stage.

Table 2 shows the effects of different water and nitrogen treatments on physiological indexes (the SPAD (Soil and Plant Analyzer Development, chlorophyll), net photosynthetic rate, transpiration rate, and stomatal conductance) in peanuts. The results show that the effect of the irrigation volume (W) on just the physiological index factors at the flowering–pegging, pod-setting, and pod-filling stages was extremely significant (*p* ≤ 0.01). Nitrogen (N) applications had highly significant (*p* ≤ 0.01) one-way effects on the SPAD, the net photosynthesis rate, and the transpiration rate at the flowering–pegging, pod-setting, and pod-filling stages and on the stomatal conductance at the pod-setting and pod-filling stages. The effect of this factor alone on the stomatal conductance at the flowering–pegging stage was significant (*p* ≤ 0.05). The interaction between the two (W × N) had highly significant effects on the SPAD, net photosynthetic rate, and stomatal conductance at the pod-setting and pod-filling stages (*p* ≤ 0.01) and on the transpiration rate at the pod-filling stage (*p* ≤ 0.01). There was a significant effect on the SPAD at the flowering–pegging stage and on the transpiration rate at the pod-setting stage (*p* ≤ 0.05).

As fertility progressed, the SPAD increased and then decreased. Its performance pattern was consistent with the LAI, indicating that excessive irrigation would reduce SPAD within a specific N application range. At the flowering–pegging stage, the net photosynthetic rate, transpiration rate, and stomatal conductance were highest, followed by at the pod-setting stage, and at the pod-filling stage, they were the lowest. When the irrigation volume increased at the N_3_ level, the peanut leaf photosynthetic rate, transpiration rate, and stomatal conductance increased, and the physiological indexes reached their maximum values. As a result of low nitrogen, the peanut leaf photosynthetic rate, transpiration rate, and stomatal conductance showed W_3_ > W_2_ > W_1_, and the physiological indexes reached their maximum value under W_3_N_1_. Peanut photosynthesis is inhibited via excessive irrigation and nitrogen fertilization. Appropriate irrigation and nitrogen application improve the peanut leaf net photosynthetic rate, transpiration rate, and stomatal conductance.

### 2.2. Effects of Water and Nitrogen Regulation on Peanut Yield under Mulched Drip Irrigation

Table 3 shows the effects of different water and nitrogen treatments on the peanut yield and yield components in the experiment. The one-way effects of water irrigation (W) on both the peanut yield and yield components were highly significant (*p* ≤ 0.01). The one-way effect of nitrogen (N) application was highly significant (*p* ≤ 0.01) on the pod yield, 100-pod weight, number of 500 g pods, and 100-kernel weight. The two interactions (W × N) had a highly significant effect (*p* ≤ 0.01) on the pod yield and a significant effect (*p* ≤ 0.05) on the 100-kernel weight.

A drip irrigation system under membrane conditions can directly impact the peanut root zone’s soil-wetting range and soil water content in the wet body, which impact the peanuts’ distribution and growth. Moreover, the development of the peanut root system affects the absorption and utilization of water and nutrients by the peanut plant. The peanut growth aboveground, and the distribution of photosynthetic products to various organs, are affected by this factor. This ultimately impacts peanuts’ economic yield. Table 3 shows that the irrigation water and nitrogen applications increased the peanut pod yield. W_3_ yielded substantially higher yields than W_2_ and W_1_. Furthermore, the N_2_ treatment yielded substantially superior results compared to the N_0_ and N_1_ treatments. The performance was consistent with the 100-pod weight, kernel rate, 100-kernel weight, pods per plant, and pod-weight-per-plant values and was negatively correlated with the 500 g pod number. The W_3_N_2_ treatment significantly increased the peanut pod yield by 92.73% and 17.13% compared with the W_1_N_2_ and W_2_N_2_ treatments under the same nitrogen application conditions. The W_2_N_2_ treatment had a 64.55% higher mortality rate than the W_1_N_2_ treatment. The W_3_N_1_ treatment significantly increased the peanut pod yield by 108.89% and 27.03% compared with the W_1_N_1_ and W_2_N_1_ treatments, and the W_2_N_1_ treatment was higher than the W_1_N_1_ treatment by 64.44%. This indicates that under drought stress, a peanut plant’s nutrient uptake capacity is severely limited. Water-deficit irrigation may lead to impaired photosynthesis in peanuts, resulting in a reduction in growth indexes, such as the main stem height and shoot dry mass, which may affect the peanut yield. Reasonable irrigation increases the soil aeration of peanut roots, promotes the respiratory function of the root layer, facilitates the absorption of soil nutrients by roots, and promotes the progression of growth indicators such as the main stem height and shoot dry mass. Therefore, reasonable irrigation is critical to peanut yield formation. The peanut pod yields under the same irrigation conditions were significantly higher in the W_3_N_2_ treatment than in the W_3_N_1_ treatment, by 12.77%; they were 22.30% and 33.09% higher in the W_2_N_2_ treatment than in the W_2_N_1_ and W_2_N_0_ treatments; 8.82% higher in the W_2_N_1_ treatment compared with the W_2_N_0_ treatment; and 22.22% higher in the W_1_N_2_ treatment compared with the W_1_N_1_. No nitrogen applications and too few nitrogen applications result in insufficient nutrients and destroy soil microecosystems. This affects crop growth indicators and yield reductions. Through applying nitrogen properly, it is possible to maintain good nutrient transport from sources (stems and leaves) to sinks (pods) and increase yields.

The relationship between peanut growth indicators and pod yield and yield components (Table 4) showed that growth indicators were closely related to the pod yield. The growth indexes were positively correlated with the pod yield, 100-pod weight, kernel rate, 100-kernel weight, pods per plant, and pod weight per plant, and were negatively correlated with the number of 500 g pods. The correlation coefficients of the growth indexes with the pod yield reached highly significant levels. Therefore, a high peanut yield was favored via increasing the main stem height, the first branch length, the branch number, the shoot dry mass, and the LAI in this experiment.

### 2.3. Effects of Water and Nitrogen Regulation on Water Consumption of Peanuts under Mulched Drip Irrigation

Table 5 shows the effects of different water and nitrogen treatments on the peanut water consumption. Peanut plants under drip irrigation with different water and nitrogen treatments consumed more water as the irrigation increased. As nitrogen applications increased, the irrigation decreased slightly. In all treatments, peanuts consumed more water during their reproductive period than during irrigation. The difference in water consumption was mainly due to irrigation. The peanuts’ water consumption was not significantly affected by nitrogen applications. As the W_3_N_2_ and W_3_N_1_ treatments had sufficient water supplies, their water consumption was the highest. Under the same nitrogen application level, the total water consumption of the W_3_N_2_ treatment during the reproductive period was 25.28% and 11.71% higher than that of W_1_N_2_ and W_2_N_2_, respectively. The W_2_N_2_ treatment consumed 12.15% more water than W_1_N_2_, the W_3_N_1_ treatment used 23.59% and 11.09% more than W_1_N_1_ and W_2_N_1_, and the W_2_N_1_ treatment used 11.25 percent more than W_1_N_1_. As a result of the differences in soil water content and irrigation water, the W_1_N_2_ and W_1_N_1_ treatments supplied less water and had the lowest soil water content control. The two treatments consumed the least amount of water, while the other treatments fluctuated between these treatments. The total water consumption in the W_3_N_1_ treatment at the same irrigation level and under different N application conditions was 1.25% higher than in W_3_N_2_; the total water consumption in the W_2_N_2_ treatment was 1.78 and 3.26% lower than that in W_2_N_1_ and W_2_N_0_; the total water consumption in W_2_N_1_ was 1.50% lower than that in W_2_N_0_; and the total water consumption in W_1_N_1_ was 2.64% greater than that in W_1_N_2_. During peanut fertility, the water consumption was significantly higher than the irrigation. Increasing the nitrogen application had little effect on the peanut water consumption, which suggests that irrigation caused most of the water consumption differences.

In drip-irrigated peanuts under different water and nitrogen treatments, the stage-wise water consumption increased with irrigation, and the differences were significant at the flowering–pegging, pod-setting, and pod-filling stages. In the sowing–emergence stage, peanuts consume the least water because of the evaporation from the bare land between films. Because peanut seedlings are short plants, with few leaves and little transpiration, the peanut water consumption is low at this stage. The peanut growth transitions from vegetative to reproductive after entering the flowering–pegging stage. In our study, the peanut plants grew rapidly, transpiration increased rapidly, and water consumption grew significantly. In the pod-setting stage of reproductive and vegetative growth, the peanut water consumption peaked. Subsequently, because of peanut maturity and temperature fall, the peanut water consumption gradually decreased.

The water consumption modulus of the seedling stage was 11.88~15.02%, that of the flowering–pegging stage was 30.20~32.99%, that of the pod-setting stage was 40.99~43.81%, and that of the pod-filling stage was 11.69~13.49%. Given the daily water consumption intensity of the peanuts in each treatment, the daily water consumption intensity was lower at the seedling stage because of the smaller plants and lower temperature, and the value was 1.68~1.85 mm·d^−1^; the growth began to transition from vegetative growth to reproductive growth at the flowering–pegging stage, with an increase in temperature; the transpiration of leaves and plants increased rapidly; and the daily water consumption intensity increased, which was between 3.53 and 4.37 mm·d^−1^. In the pod-setting stage, the plant entered the peak period, and the daily water consumption intensity reached a high value, between 3.56 and 4.82 mm·d^−1^. In the pod-filling stage, the water consumption intensity was lowest, between 0.87 and 1.21 mm·d^−1^.

### 2.4. Effects of Water and Nitrogen Regulation on Water and Nitrogen Use Efficiency of Peanuts under Mulched Drip Irrigation

Table 3 shows that the WUE (water use efficiency) was significantly higher (*p* ≤ 0.05) in the N_2_ treatment than in the N_1_ treatment at the W_1_ and W_3_ irrigation levels. The WUE was significantly higher in the N_2_ treatment than in the N_0_ and N_1_ treatments at the W_2_ irrigation level (there was no significant difference between the WUE in the N_0_ treatment and that in the N_1_ treatment). Overall, it appeared that irrigation and fertilizer application had a highly significant (*p* ≤ 0.01) effect, either singly or interactively, on both the WUE and IWUE (irrigation water use efficiency).

The NPP (nitrogen partial factor productivity) is an index reflecting the comprehensive effect of local soil basic nutrient levels and the chemical fertilizer application rate. The NPP was inversely proportional to the amount of fertilizer applied at the same irrigation level. As the N applications grew, the NPP decreased significantly. The NPP gradually increased as the irrigation water became plentiful. The NPP in the W_3_N_2_ treatment was substantially higher than those in the W_1_N_2_ and W_2_N_2_ treatments at the N_2_ level (*p* ≤ 0.05), and the W_3_N_1_ and W_2_N_1_ treatments were considerably better than the W_1_N_1_ treatment at the N_1_ level (*p* ≤ 0.05). Overall, it appeared that both the irrigation and fertilization rates had highly significant (*p* ≤ 0.01) effects on the NPP, either singly or interactively (*p* ≤ 0.01). Therefore, increasing the irrigation volume simultaneously with the N applications could ensure a higher NPP. In this experiment, the NPP was highest in the W_3_N_1_ treatment at 60.61 kg·kg^−1^, followed by in the W_3_N_2_ treatment at 48.16 kg·kg^−1^, which differed significantly from the other treatments.

### 2.5. Based on a Comprehensive Evaluation, the Most Appropriate Irrigation and Nitrogen Application Indexes Were Selected

The pod yield (x_1_), the 100-pod weight (x_2_), the 500 g pod numbers (x_3_), the kernel rate (x_4_), the 100-kernel weight (x_5_), the pod per plant (x_6_), the pod weight per plant (x_7_), the WUE (x_8_), the IWUE (x_9_), and the NPP (x_10_) of peanuts under different water and nitrogen treatments were analyzed via correlation, principal component, and membership function analyses (Table 6, Table 7, Table 8 and Table 9). The results showed (Table 6) that the correlation coefficients between the indicators of peanuts reached highly significant levels (*p* ≤ 0.01). This shows that it is crucial to coordinate source–sink translocation to obtain optimal peanut yields, and that all high-yielding peanuts are characterized by a high water and nitrogen use efficiency.

The membership function method can comprehensively evaluate the peanut indicator stability. The larger the positive value of an indicator (or the smaller the negative value), the smaller the range, the larger the membership function value R, and the more stable the indicators. The results show that (Table 7) the membership function values of different treatments were the highest in the W_3_N_2_ treatment, and the indicators were stable.

Principal component analysis was conducted on the 10 peanut indexes, and the initial characteristics of each extracted component were determined (Table 8). The results showed that the cumulative contribution rate of 10 peanut components reached 100%, indicating that 10 factors contributed to the comprehensive peanut evaluation. The cumulative contribution rate of the first two principal components reached 89.733%, which contained all the variation information from the original variables. Choosing the first two principal components as the main principal components allowed us to reduce the number of variables and retain most of the original information.

The principal component matrix reflects the direction of the relative magnitude and the effect of the main index on the principal component load, that is, the degree to which the index influences the principal component, also referred to as the index load on the principal component (Table 9). Based on the matrix coefficients and normalized data, the score function expressions for the two principal components can be obtained:

Z_1_ = 0.341x_1_ + 0.329x_2_ − 0.334x_3_ + 0.298x_4_ + 0.333x_5_ + 0.300x_6_ + 0.296x_7_ + 0.332x_8_ + 0.316x_9_ + 0.278x_10_;

Z_2_ = −0.050x_1_ − 0.1093x_2_ + 0.144x_3_ − 0.506x_4_ − 0.061x_5_ + 0.417x_6_ + 0.366x_7_ + 0.198x_8_ + 0.341x_9_ − 0.495x_10_.

Based on the variance contribution ratio of each principal component, the comprehensive evaluation function can be derived: F = 0.8399Z_1_ + 0.5744Z_2_.

According to the comprehensive evaluation score (Table 10), the highest comprehensive score treatment was W_3_N_2_, and the lowest treatment was W_1_N_1_. Therefore, the water and nitrogen regulation treatment with the highest yield was W_3_N_2_.

## 3. Discussion

### 3.1. Effects of Water and Nitrogen Regulation on Growth and Physiological Indexes of Peanuts under Mulched Drip Irrigation

In Xinjiang, peanuts now have better development prospects and can be developed into a new, advantageous oilseed resource. However, they require water and fertilizers to grow. Water [21] and nitrogen [22,23] are key factors affecting peanut growth and development. Appropriate water and nitrogen regulation can promote peanut nutritional and reproductive efficiency. According to Longnecker [24], Rodriguez [25], and other researchers’ studies, nitrogen fertilization benefits crop nutrient organs and the development of plant population indexes, resulting in improved plant growth. At the same time, it can improve the unfavorable effects of water stress conditions, such as slow leaf extension and a reduced leaf area; increase crop dry matter accumulation; and reduce yield losses. Qi et al. [26] showed that nitrogen fertilizer application under water adversity conditions had a significant regulatory effect on winter wheat plant growth and dry matter accumulation. In addition, water deficits during reproductive periods affected the plant height, leaf area, and dry matter accumulation. A study by Liu et al. [27] indicated that, with an increase in irrigation and fertilizer application, the seedling height, leaf area, and dry matter mass increase in small-grain coffee, a finding that seems to align with this paper’s results. This study showed that increasing irrigation at N_0_, N_1_, and N_2_ benefits the development of growth indicators. This indicates that water positively affects peanut growth, adequate water supply, crop absorption, and utilization. A significant increase in the main stem height reveals this. We also concluded that the peanuts’ vegetative growth was inhibited by low nitrogen (N_1_) levels and the absence of nitrogen fertilizer (N_0_). Peanuts treated with N_2_ experienced significant growth, improved quality, and yield increases.

Plant growth is governed by the physiological indicators of leaves, which determine how much dry matter accumulates and how high a yield is produced [28,29]. Photosynthetic products account for 90~95% of dry matter accumulation. At different fertility stages, nitrogen and water regulation will differentially affect crop-leaf SPAD values, net photosynthetic rates, transpiration rates, and stomatal conductance. According to Li et al. [30], cotton leaves’ relative SPAD, net photosynthetic rate, and maximum photochemical efficiency decrease under drought stress. Su et al. [31] concluded that regulated deficit irrigation decreases alfalfa’s photosynthetic rate and reduces light energy utilization. Stomatal closure affects photosynthetic product accumulation in the plant. Shen et al. [32] found that water stress decreases the net photosynthetic rate of crop leaves, the production of photosynthetic products, and the physiological activity of various organs, thereby limiting the plant’s ability to absorb nutrients and accumulate them and affecting its economic yield. During the reproductive period of the study, the physiological indexes (the SPAD value, net photosynthetic rate, transpiration rate, and stomatal conductance) in the low-water treatment were significantly lower than those in the high-water treatment; i.e., because of the water deficit, the peanuts’ photosynthesis was suppressed, resulting in lower physiological index values, and the values of physiological indexes increased as irrigation increased, indicating that irrigation had a stronger compensatory effect on the plants. Consequently, water control is the key to crop photosynthesis, and moderate water treatment can significantly improve peanut physiological indexes. Li et al. [33] found that a moderate increase in nitrogen fertilization during drought stress increases the chlorophyll content of cotton’s primary stem leaves and net photosynthetic rate, and it mitigated the drought stress inhibition of photosynthesis. According to that study, peanuts benefit from nitrogen fertilizer through maintaining high physiological indexes. Plants allocate nutrient resources to the organs or parts of their bodies that need that nutrient most, which means that their photosynthesis characteristics are affected when nitrogen is reduced, so they can satisfy their growth, development, and physiological metabolism. If nitrogen fertilizer is applied too much, the leaves will shade one another, and the light conditions will become worse in the lower part of the plant, accelerating the senescence process of the lower leaves and decreasing the nitrogen content of the lower part of the plant, and thereby adversely affecting photosynthetic production. All the physiological indexes of the peanuts increased with an increase in nitrogen application over their entire life span. However, the maximum index under N_2_ treatment did not appear to decline, which indicates that the nitrogen application in the N_2_ treatment did not reach its maximum value, and a higher-gradient nitrogen application should be used in future studies. In summary, irrigation and nitrogen applications can facilitate peanut photosynthesis and dry matter conversion.

### 3.2. Effects of Water and Nitrogen Regulation on Peanut Yield under Mulched Drip Irrigation

Crop yields are heavily influenced by moisture and nitrogen [34]. By transporting nutrients and promoting nitrogen conversion, moisture can regulate soil moisture and promote water absorption in plants [35]. According to an in-depth study of the effects of water and fertilizer on plants, “fertilizer regulates water, water enhances fertilizer” [36]. In this study, nitrogen fertilizer increased the peanut yield and yield components compared with the non-application of nitrogen fertilizer. Cong et al. [37] demonstrated that moderate irrigation can improve yield factors, excessive irrigation plays an inhibitory role, yield and nitrogen applications are not always correlated positively, excessive nitrogen applications will reduce yield, and only appropriate irrigation and nitrogen applications can improve wheat yield. The results of this study show that, at the same irrigation level, increasing nitrogen fertilizer can increase peanut pod yields, 100-pod weights, the number of 500 g fruits, kernel rates, 100-kernel weights, pods per plant, and pod weights per plant. All of these reached their maximum in the W_2_ treatment; under the same level of nitrogen application, increasing the amount of irrigation can increase the indexes, which is inconsistent with the results of the above study and may be due to the maximum amount of irrigation being not enough; thus, the results of the current experiment will be supplemented to verify the results of subsequent experiments. In this study, a continuous upward trend in peanut yield was observed with an increase in nitrogen applications. An in-depth study of the proportion of water and nitrogen inputs and peanut production is indispensable in achieving high-yield and water- and fertilizer-saving peanut cultivation.

### 3.3. Effects of Water and Nitrogen Regulation on the Water Consumption of Peanuts under Mulched Drip Irrigation

In northern Xinjiang, the peanuts under membrane drip irrigation experienced an increase in temperature over the entire growth period. With a temperature increase, the peanuts’ water consumption increased gradually, and they required about 402.57 mm of water to reach a high yield. In this study, peanut pods consumed the most water during the pod-setting stage. This is when the embryonic and nutrient bodies grew simultaneously, and is a critical period for peanut pods to fill up and become complete. The water requirements of peanut plants vary from reproductive stage to reproductive stage. Feng et al. [38] showed that, with sprinkler irrigation, the peanuts in their study consumed the most water during the flowering–pegging and pod-setting stages, and the average daily water consumption decreased as the sprinkler irrigation was reduced. In contrast, the opposite was confirmed for whole fruit. In their study of peanut plant water consumption, Xia et al. [39] found that water consumption increased with irrigation quotas during a peanut’s development. The peanuts’ water consumption pattern showed a lesser consumption at the end. As with the study in this paper, an extensive irrigation quota resulted in a relatively high water consumption and intensity. According to Ren et al. [40], peanut yield is closely related to water consumption, the peanut yield improved as the irrigation water increased, the water consumption grew to about 650 mm, the yield declined, and the overall trend was parabolic. This experiment is a preliminary study of under-membrane drip irrigation peanuts. Our water consumption values did not reach the threshold value, but we will increase it in next year’s trial.

### 3.4. Effects of Water and Nitrogen Regulation on Water and Nitrogen Use Efficiency of Peanuts under Mulched Drip Irrigation

Sustainable agricultural development requires the improvement of water and fertilizer efficiency and the enhancement of crop yields. According to Li et al. [41], enhancing nitrogen application improves crops’ water use efficiency under the same irrigation conditions. The WUE is significantly affected by irrigation and nitrogen applications, as reported by Kaisi et al. [42]. In our study, the WUE and IWUE showed increasing trends with increasing N applications and reached their maxima at N_2_. When comparing each irrigation level under this N application condition, we can see that the WUE and IWUE were at their maxima in W_3_N_2_. The yield decreases the water consumption and efficiency, according to Huang et al. [43]. With a 5.2974 Mg·ha^−1^ yield, the W_3_N_2_ treatment showed the highest water use efficiency. Given the water–nitrogen coupling effect in this experiment, irrigation increased the nitrogen fertilizer efficacy. However, the increased application of nitrogen fertilizer contributed to a higher pod yield, resulting in a higher water use efficiency.

The nitrogen fertilizer bias productivity decreases and improves with increasing nitrogen applications and irrigation, respectively, according to Aujla et al. [44]. As in the previous study, the NPP decreased with increasing N applications under the same irrigation conditions. In low-nitrogen conditions, the nitrogen bias productivity was highest with the high-water treatment. Irrigation has a significant nitrogen-promoting effect in arid regions, and the nitrogen fertilizer’s water-promoting effect is more apparent only when the soil is moist, so moisture is the decisive factor for boosting peanut yields during under-membrane drip irrigation.

Under-membrane drip irrigation was evaluated using principal component analysis to comprehensively evaluate the peanut yield, yield components, and water and nitrogen utilization efficiency. According to the principal component analysis, the W_3_N_2_ treatment had the highest comprehensive score, and the yield was significantly higher than that in the other treatments. Overall, the W_3_N_2_ treatment significantly increased the yield and proved to be a better water–nitrogen combination. Furthermore, the irrigation volume in this trial was low overall, and the optimal nitrogen application level was lower than that in other studies. In subsequent experiments, the irrigation volume should be increased to allow us to study water–nitrogen interactions.

## 4. Materials and Methods

### 4.1. Overview of the Test Area

**Figure 1:**
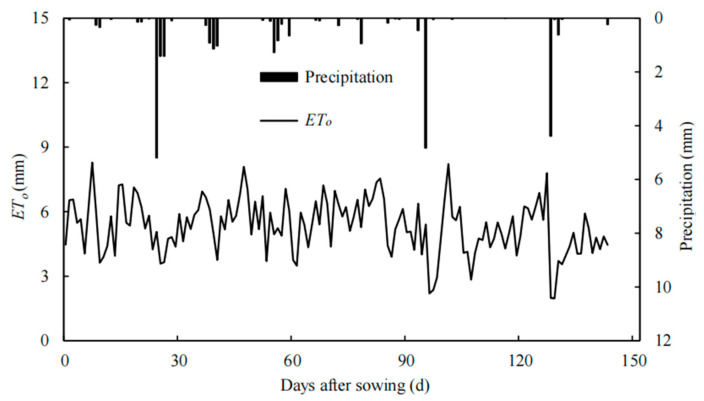
Precipitation and *ET_o_* in the peanut growth period under mulched drip irrigation in Xinjiang.

We experimented at the Xinjiang Academy of Agricultural Sciences, Anning Canal (87°30′ E, 43°58′ N, 590 m above sea level) from May to October 2022. The experimental base is located in Urumqi City, Xinjiang Uygur Autonomous Region, in the middle of the northern foothills of the Tianshan Mountains and the northern outskirts of Urumqi City on the alluvial plains. The area has a gentle terrain, which is typical of the economic belt on the northern slopes of the Tianshan Mountains. The average annual sunshine hours in the test area are 2700–2800 h, the effective accumulated temperature greater than 10 °C is 3000–3500 °C [1], the frost-free period is 170~179 d, the annual average precipitation is 200 mm, the maximum evapotranspiration is 1750 mm, the groundwater depth is 7.5 m, and it is located in the arid and semi-arid desert climatic zone agricultural area. In the experimental area, the pH of the tillage soil ranged from 7.8 to 8. The physical and chemical properties of the 0–60 cm soil layer are presented in Table 11. Figure 1 shows the precipitation and *ET_o_* (reference evapotranspiration) values for the experimental period. The reference crop evapotranspiration (*ET_o_*) was calculated using the Penman–Monteith formula [45].

### 4.2. Peanut Cultivation Patterns

**Figure 2:**
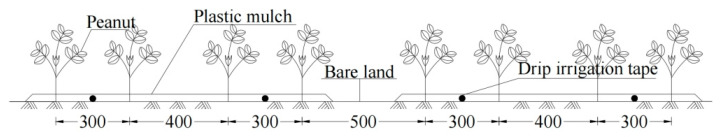
Layout of drip irrigation in the peanut field under film mulching (mm).

In a membrane drip irrigation peanut planting mode, with one membrane and four rows (Figure 2), the average hole spacing was 15 cm, and the planting density was 166,000 holes per hectare. Sowing began on 7 May (dry sowing and wetting out); harvesting began on September 27 from whole seedlings. Before sowing, the basal compound fertilizer (N: P_2_O_5_:K_2_O = 15%:15%:15%) was 300 kg·ha^−1^, and nitrogen fertilizer (urea (CO(NH_2_)_2_, nitrogen content ≥ 46%)) was applied four times during the reproductive period via fertilizing with water. Table 12 shows the amount of nitrogen fertilizer (pure nitrogen) for each treatment. The chemical control, spraying, and other agronomic measures were the same as in the high-yield farmland management model. The irrigation system used a sub-film drip irrigation system with labyrinth drip tape for the capillary, a 3.2 L·h^−1^ drip head flow rate, and 30 cm drip-head spacing. There were five plots for each treatment (the length and width of each plot were 54 m and 1.5 m, respectively) on a 1620 m^2^ experimental area controlled by a branch pipe. This branch pipe irrigation system had gate valves and water meters at its entrance. Groundwater provided irrigation water, and the meters controlled how much irrigation water was applied.

### 4.3. Experimental Design

Peanuts (Huayu 9610, registration number: GPD Peanuts (2018) 370377 [46]) were tested. The peanut growth was divided into five stages based on growth stages: the seedling stage (from the emergence of the first true leaves on 50% of seedlings in the peanut field to the opening of the first flowers on 50% of the plants in the peanut field), the flowering–pegging stage (from the opening of the first flowers on 50% of the plants in the peanut field to the emergence of the first chicken-headed young pods on 50% of the plants in the peanut field), the pod-setting stage (from the emergence of the first chicken-headed young pods on 50% of the plants in the peanut field to the emergence of whole pods on 50% of the plants in the peanut field), the pod-filling stage (from 50% of the plants in the peanut field to 100% of the plants, the pods are whole and mature), and the harvest stage. This was a split-area trial with two factors: water and nitrogen. The nitrogen fertilizer dosage was set at two levels: 77.5 and 110 kg·ha^−1^. With a total of seven treatments (Table 12), the irrigation quota was set at three levels of 22.5, 30, and 37.5 mm, and there was a complete combination of two factors, six treatments, and a control treatment without nitrogen applications (W_2_N_0_). Each treatment was replicated five times. Peanuts are not irrigated during seedling and harvesting, according to local production practices. During the 2022 peanut growing season, 45 mm was used for irrigation to ensure peanut emergence after sowing. The actual irrigation and fertilization times, irrigation volumes, and nitrogen application rates during the peanut growth period are shown in Table 13.

### 4.4. Observation Items and Methods

#### 4.4.1. Soil Moisture Content

Before sowing, irrigation, and harvest, the soil gravimetric method was used to measure the moisture within the layers (0–20, 20–40, 40–60, 60–80, 80–100 cm) and, after irrigating within the layers (0–10, 10–20, 20–30, 30–40, 40–50, 50–60 cm), we took into account the cultivation characteristics of the wide and narrow rows of peanuts and the drip irrigation line infiltration characteristics. Each treatment was sampled four times at 0 cm (in the film), 20 cm (in the inner row), 35 cm (under the drip irrigation belt), and 70 cm (between the films); in each treatment, a sample point was selected directly below the drip irrigation belt before sowing, before irrigation, and after harvest, and the average soil water content was determined using the Shen method [47] to represent the average soil water content in the peanut fields.

#### 4.4.2. Measurement of Crop Growth and Physiological Indexes

A straightedge was used to measure the height of the main stem and first branch length during the reproductive period and to investigate the number of branches.

For the shoot dry matter, destructive sampling was carried out at the main fertility stage of the peanuts, with three randomly collected plant samples from each plot placed in an oven at 105 °C for half an hour for the killing process, followed by the peanut samples being dried to a constant weight at a constant temperature of 85 °C.

The leaf area index (LAI) was measured using the specific leaf weight method during the main fertility period.

The chlorophyll content of the leaves was measured using a SPAD-502Plus Chlorophyll Meter.

Regarding the net photosynthetic rate, the transpiration rate and stomatal conductance of the peanut plants were measured using Li-6800 portable photosynthesis systems (Li-COR Inc., Lincoln, NW, USA) on three randomly selected peanut plants in each plot between 9:00 and 13:00 on a clear day.

#### 4.4.3. Calculation of Field Yield and Water Consumption

We evaluated the pod yield, the 100-pod weight, the 100-kernel weight, the 500 g pod count, and the kernel rate at the ripe harvest stage using 6.67 m^2^ per treatment as the unit yield.

Equation (1) was used to calculate the water consumption in each treatment using the water balance equation [45]. As the buried depth of groundwater in the test area was greater than 7.5 m, the effect of groundwater recharge could be ignored. *K* = 0 as there was no adequate precipitation of over 10 mm during the whole peanut growth stage, and *P*_0_ = 0. In the test field [48], irrigation water impacted the surface by 60 mm when the irrigation quota was less than 37.5 mm. *D* = 0 if the irrigation rate was less than 37.5 mm in the drip-irrigated peanut fields. Equation (1) can be simplified into Equation (2):

(1) $$\mathrm{ET}=P_{0}+K+M-D+(W_{0}-W_{t})$$

(2) $$\mathrm{ET}=M+(W_{0}-W_{t})$$

where *ET* is the peanut water consumption (mm); *P*_0_ is adequate precipitation (mm); *K* is the groundwater recharge (mm); *M* is the irrigation water (mm); *D* is the deep seepage (mm); and *W*_0_ and *W_t_* denote the soil water storage at the beginning and the end of the period.

#### 4.4.4. Water Use Efficiency

The water use efficiency of a crop refers to its level of photosynthesis or growth per unit of evapotranspiration, that is, the amount of financial product produced per unit of water. A crop’s water use efficiency is evaluated using this indicator. Under drip irrigation conditions, Equations (3) and (4) can be used to calculate the irrigation water use efficiency (*IWUE*) of different water treatments:

(3) $$\mathrm{WUE}=Y/ET_{a}$$

(4) $$\mathrm{IWUE}=Y/ET_{a}$$

where *WUE* and *IWUE* denote the water use efficiency and irrigation water use efficiency (kg·m^−3^), respectively; *Y* is the pod yield of peanuts (kg·ha^−1^); *ET_a_* is the actual water consumption of peanuts during the whole growth stage (m^3^·ha^−1^); and *I* is the total amount of irrigation during the whole growth stage of peanuts during under-membrane drip irrigation, i.e., the irrigation quota (m^3^·ha^−1^).

#### 4.4.5. Nitrogen Partial Factor Productivity


(5) NPP=Y/N

Here, *NPP* represents the nitrogen partial factor productivity (kg·kg^−1^); and *N* is the total amount of nitrogen applied to peanuts during under-membrane drip irrigation throughout the growth stage (kg·ha^−1^).

### 4.5. Comprehensive Evaluation

#### 4.5.1. Membership Function Method

The pod yield, 100-pod weight, number of 500 g fruits, kernel rate, 100-kernel weight, WUE, and NPP were comprehensively evaluated using membership function analysis. The subordination function value of each indicator was calculated as follows:

(6) $$R_{\mu 1}=\frac{x-x_{\min}}{x_{\max}-x_{\min}}$$

(7) $$R_{\mu 2}=1-\frac{x-x_{\min}}{x_{\max}-x_{\min}}$$

where *R* is the measured value of the measured index of a certain treatment, *μ*_1_ indicates the forward index, and *μ*_2_ indicates the reverse indicator. *x_min_* and *x_max_* denote the minimum and maximum values of the measured indexes in all treatments, respectively. If the measured indicator is a positive indicator, Formula (6) is used to calculate the membership function value of the indicator; otherwise, Formula (7) is used.

#### 4.5.2. Principal Component Analysis

Principal component analysis is based on data dimension reduction. It involves using lossless information to multiply indicators in linear combinations into a few related comprehensive indicators using a multivariate statistical analysis method; its main purpose is to reduce the data dimension to eliminate overlapping and coexisting information. The index should be simplified as much as possible to reflect the main information of the original index.

Let the original matrix observation data be set as follows:

(8) $$X\;=\left[\begin{array}{cccc} x_{11} & x_{12} & \cdots & x_{1p}\\ x_{21} & x_{22} & \cdots & x_{2p}\\ \vdots & \vdots & \vdots & \vdots \\ x_{n1} & x_{n2} & \cdots & x_{\mathrm{np}} \end{array}\right]$$

The normalized matrix was obtained via the original matrix normalization process:

(9) $$x_{ij}^{*}=\frac{x_{ij}-{\bar{x}}_{j}}{S_{j}}\;i=1,2,\ldots ,n;j=1,2,\ldots ,p$$

(10) $${\bar{x}}_{j}=\frac{1}{n}\sum_{i=1}^{n}x_{\mathrm{ij}}$$

(11) $$S_{j}^{2}=\frac{1}{n\;-1}\sum_{i=1}^{n}{{(x}_{\mathrm{ij}}-{\bar{x}}_{j})}^{2}$$

(12) $$X^{*}=\left[\begin{array}{cccc} x_{11}^{*} & x_{12}^{*} & \cdots & x_{1p}^{*}\\ x_{21}^{*} & x_{22}^{*} & \cdots & x_{2p}^{*}\\ \vdots & \vdots & \vdots & \vdots \\ x_{n1}^{*} & x_{n2}^{*} & \cdots & x_{\mathrm{np}}^{*} \end{array}\right]$$

The correlation coefficient matrix is calculated as follows:

(13) $$R^{x}=\left[\begin{array}{cccc} r_{11} & r_{12} & \cdots & r_{1p}\\ r_{21} & r_{22} & \cdots & r_{2p}\\ \vdots & \vdots & \vdots & \vdots \\ r_{n1} & r_{n2} & \cdots & r_{\mathrm{np}} \end{array}\right]$$

(14) $$r^{\mathrm{ij}}=\frac{1}{n\;-1}\sum_{i=1}^{n}x_{\mathrm{ti}}x_{\mathrm{tj}}\;i=1,2,\ldots ,p;\;j=1,2,\ldots ,p$$

We calculated the eigenvalues of the correlation coefficient matrix (λ_1_, λ_2_, λ) and the corresponding eigenvectors (a*_i_*_1_, a*_i_*_2_, …, a*_ip_*).

We calculated the contribution rate of each principal component:

(15) $$C_{r}=\frac{\lambda_{i}}{\sum_{i=1}^{n}\lambda_{i}}\;i=1,2,\ldots ,p$$

According to the normalized matrix data, the principal component expression is the final score of the principal component.

### 4.6. Meteorological Indicators

The Chinese Meteorological Data Service Center (http://data.cma.cn/, format: accessed on 21 March 2023) provided selected meteorological data on the temperature, solar radiation, wind speed, relative humidity, and precipitation during the peanut growth stages.

### 4.7. Data Analysis

The experimental data were collected and analyzed, and graphs were plotted, using Excel 2016. In this study, multiple comparisons were performed using the Duncan new compound difference method analysis. Significant differences between the detected parameters were compared using Tukey’s honest significant difference (HSD) test at the 95% confidence level (*p* ≤ 0.05).

## 5. Conclusions

(1) The daily water consumption intensity of peanuts ranged from 1.68 to 1.85 mm·d^−1^ at the seedling stage and from 3.53 to 4.37 mm·d^−1^ at the flowering–pegging stage. It reached a maximum of 3.56–4.82 mm·d^−1^ at the pod-setting stage and decreased from 0.87 to 1.21 mm·d^−1^ at the pod-filling stage.

(2) Compared with no N application (N_0_), an increased N application significantly improved physiological indices, promoted peanut growth indices and, therefore, increased yield. The NPP decreased with increased N application.

(3) Based on the comprehensive evaluation, under the conditions of this experiment, a higher peanut yield of 5.2974 Mg·ha^−1^; a higher WUE of 1.32 kg·m^−3^ at an irrigation quota of W_3_ (37.5 mm); an application rate of N_2_ (110 kg·ha^−1^); and a watering cycle of 10–15 d are the appropriate water and nitrogen regulation values for peanut production during drip irrigation under membrane conditions in Xinjiang.

## Figures and Tables

**Table 1:** Effects of water and nitrogen regulation on the growth traits of peanuts under mulched drip irrigation in Xinjiang.

Treatment	Main Stem Height (cm)				First Branch Length (cm)				Branch Number (pcs·plant^−1^)				Shoot Dry Mass (kg·ha^−1^)				LAI			
	Seedling Stage	Flowering–Pegging Stage	Pod-Setting Stage	Pod-Filling Stage	Seedling Stage	Flowering–Pegging Stage	Pod-Setting Stage	Pod-Filling Stage	Seedling stage	Flowering–Pegging Stage	Pod-Setting Stage	Pod-Filling Stage	Seedling Stage	Flowering–Pegging Stage	Pod-Setting Stage	Pod-Filling Stage	Seedling Stage	Flowering–Pegging Stage	Pod-Setting Stage	Pod-Filling Stage
W_1_N_2_	3.23 bc	11.60 abc	16.47 c	19.53 d	3.20 b	12.03 ab	17.37 bc	23.50 c	8.67 a	12.67 ab	13.67 ab	14.67 b	752.59 a	3937.19 c	6848.20 cd	8712.30 cd	0.53 a	0.91 bc	3.85 b	4.47 cd
W_2_N_2_	3.97 ab	12.30 ab	19.17 ab	26.73 b	3.97 ab	13.13 a	18.33 b	33.10 a	7.67 a	12.67 ab	16.00 a	17.00 a	760.89 a	4778.07 a	11,231.80 a	18,409.09 a	0.51 a	1.66 a	4.73 a	8.56 a
W_3_N_2_	2.77 bc	13.67 a	19.77 a	28.13 a	3.40 ab	13.73 a	23.67 a	33.40 a	8.67 a	13.33 a	16.00 a	16.67 a	789.93 a	4659.56 ab	8225.98 bc	11,868.84 b	0.51 a	1.14 b	4.64 a	7.34 b
W_1_N_1_	4.57 a	8.93 d	10.40 e	15.50 e	4.33 a	9.20 c	9.83 d	18.47 e	7.67 a	10.00 c	12.33 b	12.67 c	719.41 a	1598.81 e	4520.69 e	6709.33 d	0.51 a	0.74 c	1.96 d	2.88 e
W_2_N_1_	3.20 bc	10.07 bcd	16.07 c	24.13 c	4.07 ab	11.53 ab	14.93 c	26.97 b	8.00 a	12.33 ab	14.33 ab	14.67 b	685.04 a	4118.52 bc	7689.28 bc	9256.69 bcd	0.5 a	0.93 bc	4.06 ab	4.78 c
W_3_N_1_	3.50 abc	11.50 abc	18.40 b	26.10 b	4.03 ab	12.23 ab	19.93 b	27.40 b	7.33 a	12.00 b	15.00 a	15.67 ab	744.30 a	4699.85 ab	8727.51 b	10,353.78 bc	0.52 a	1.14 b	4.25 ab	5.4 c
W_2_N_0_	2.60 c	9.43 cd	15.00 d	19.07 c	3.60 ab	10.80 bc	14.77 b	20.23 d	8.00 a	11.67 b	14.00 ab	15.00 b	764.44 a	2680.89 d	6123.85 d	8255.21 cd	0.49 a	0.43 d	2.75 c	3.63 cd
W	ns	**	**	**	ns	**	**	**	ns	**	**	**	ns	**	**	**	ns	**	**	**
N	ns	**	**	**	ns	**	**	**	ns	**	*	*	ns	**	**	**	ns	**	**	**
W × N	ns	ns	**	**	ns	ns	ns	*	ns	*	ns	ns	ns	**	**	**	ns	*	*	*

**Note:** Different lowercase letters show that mean values are significantly different from one another at *p* ≤ 0.05. “ns” means not significant (*p* > 0.05); “*” means significant (*p* ≤ 0.05); “**” means extremely significant (*p* ≤ 0.01); the same is the case in the tables below.

**Table 2:** Effects of water and nitrogen regulation on the physiological indexes of peanuts under mulched drip irrigation in Xinjiang.

Treatment	SPAD				Net Photosynthetic Rate (µmol m^−2^ s^−1^)			Transpiration Rate (mmol m^−2^ s^−1^)			Stomatal Conductance (mol m^−2^ s^−1^)		
	Seedling Stage	Flowering–Pegging Stage	Pod-Setting Stage	Pod-Filling Stage	Flowering–Pegging Stage	Pod-Setting Stage	Pod-Filling Stage	Flowering–Pegging Stage	Pod-Setting Stage	Pod-Filling Stage	Flowering–Pegging Stage	Pod-Setting Stage	Pod-Filling Stage
W_1_N_2_	49.70 a	50.37 de	54.50 d	44.90 c	13.79 de	9.50 d	5.26 c	3.36 bc	2.02 d	1.73 a	0.14 b	0.07 c	0.04 b
W_2_N_2_	50.30 a	53.90 a	58.90 a	54.23 a	17.49 a	14.30 a	7.03 a	6.43 a	2.88 a	1.95 a	0.31 a	0.12 a	0.05 a
W_3_N_2_	50.77 a	52.43 b	58.03 b	47.80 b	15.47 b	10.91 c	6.75 ab	4.18 b	2.77 a	1.73 a	0.18 b	0.08 b	0.05 ab
W_1_N_1_	49.80 a	50.27 de	50.30 f	41.73 d	14.31 cd	7.81 e	1.41 d	2.16 d	1.61 e	0.37 c	0.08 b	0.04 d	0.01 d
W_2_N_1_	50.63 a	51.17 cd	55.23 c	45.30 c	14.53 bcd	9.67 d	1.86 d	5.95 a	2.31 c	0.94 b	0.28 a	0.07 c	0.02 c
W_3_N_1_	49.80 a	52.13 bc	57.83 b	47.37 b	15.08 bc	12.14 b	4.46 c	3.34 bc	2.41 b	1.81 a	0.14 b	0.08 c	0.05 ab
W_2_N_0_	49.37 a	50.00 e	51.67 e	44.07 c	13.05 e	7.05 e	5.64 bc	2.59 cd	2.14 d	1.59 a	0.13 b	0.06 c	0.05 ab
W	ns	**	**	**	**	**	**	**	**	**	**	**	**
N	ns	**	**	**	**	**	**	**	**	**	*	**	**
W × N	ns	*	**	**	ns	**	**	ns	*	**	ns	**	**

**Note:** Different lowercase letters show that mean values are significantly different from one another at *p* ≤ 0.05. “ns” means not significant (*p* > 0.05); “*” means significant (*p* ≤ 0.05); “**” means extremely significant (*p* ≤ 0.01).

**Table 3:** Effects of water and nitrogen regulation on the yield index and water use efficiency of peanuts under mulched drip irrigation in Xinjiang.

Treatment	Pod Yield (Mg·ha^−1^)	100-Pod Weight(g)	500 g Pod Number (pcs)	Kernel Rate (%)	100-Kernel Weight (g)	Pod per Plant (pcs)	Pod Weight per Plant (g)	WUE (kg·m^−3^)	IWUE (kg·m^−3^)	NPP (kg·kg^−1^)
W_1_N_2_	2.7486 d	140.82 d	373.00 b	61.76 e	63.24 de	15.67 bc	17.75 cd	0.86 d	1.06 d	24.99 e
W_2_N_2_	4.5227 b	179.42 b	301.00 cd	68.29 abc	77.44 c	19.00 b	22.84 bc	1.25 ab	1.38 a	41.12 c
W_3_N_2_	5.2974 a	208.54 a	257.67 e	70.83 a	95.42 a	24.00 a	31.14 a	1.32 a	1.41 a	48.16 b
W_1_N_1_	2.2489 d	136.58 d	407.00 a	62.93 de	58.64 e	13.00 c	14.82 d	0.68 e	0.86 e	29.02 d
W_2_N_1_	3.6982 c	165.26 c	314.33 c	66.47 bc	72.86 c	18.00 b	21.11 abc	1.01 cd	1.12 c	47.72 b
W_3_N_1_	4.6977 b	180.81 b	281.67 de	69.13 ab	85.29 b	19.67 b	25.67 ab	1.15 bc	1.24 b	60.61 a
W_2_N_0_	3.3983 c	156.48 c	350.00 b	65.61 cd	70.33 cd	17.67 b	20.43 abc	0.91 d	1.08 d	-
W	**	**	**	**	**	**	**	**	**	**
N	**	**	**	ns	**	ns	ns	**	**	**
W × N	**	*	ns	ns	ns	ns	ns	**	**	**

**Note:** Different lowercase letters show that mean values are significantly different from one another at *p* ≤ 0.05. “ns” means not significant (*p* > 0.05); “*” means significant (*p* ≤ 0.05); “**” means extremely significant (*p* ≤ 0.01).

**Table 4:** The correlation coefficients of the growth index, with the yield and yield components.

Index	Pod Yield	100-Pod Weight	500 g Pod Number	Kernel Rate	100-Kernel Weight	Pod per Plant	Pod Weight per Plant
Main stem height	0.928 **	0.902 **	−0.950 **	0.791 **	0.870 **	0.769 **	0.759 **
First branch Length	0.873 **	0.843 **	−0.842 **	0.694 **	0.785 **	0.700 **	0.691 **
Branch number	0.803 **	0.730 **	−0.712 **	0.555 **	0.695 **	0.713 **	0.598 **
Shoot dry mass	0.613 **	0.561 **	−0.538 *	0.519 *	0.427	0.426	0.336
LAI	0.735 **	0.715 **	−0.706 **	0.603 **	0.652 **	0.616 **	0.654 **

**Note:** “*” means significant (*p* ≤ 0.05); “**” means extremely significant (*p* ≤ 0.01).

**Table 5:** Effects of different treatments on the water consumption of peanuts under mulched drip irrigation in Xinjiang.

Treatment	Seedling Stage			Flowering–Pegging Stage			Pod-Setting Stage			Pod-Filling Stage			Total Growth Stage
	Water Consumption(mm)	Water Consumption Intensity (mm·d^−1^)	Water Consumption Percentage (%)	Water Consumption(mm)	Water Consumption Intensity (mm·d^−1^)	Water Consumption Percentage (%)	Water Consumption(mm)	Water Consumption Intensity (mm·d^−1^)	Water Consumption Percentage (%)	Water Consumption(mm)	Water Consumption Intensity (mm·d^−1^)	Water Consumption Percentage (%)	Water Consumption(mm)
W_1_N_2_	48.26	1.72	15.02	102.43	3.53	31.88	131.70	3.56	40.99	38.95	0.87	12.12	321.33
W_2_N_2_	51.66	1.85	14.33	114.98	3.96	31.91	151.61	4.10	42.07	42.13	0.94	11.69	360.38
W_3_N_2_	50.34	1.80	12.50	121.56	4.19	30.20	176.37	4.77	43.81	54.30	1.21	13.49	402.57
W_1_N_1_	47.09	1.68	14.28	104.86	3.62	31.79	137.81	3.72	41.78	40.05	0.89	12.14	329.81
W_2_N_1_	50.43	1.80	13.74	120.08	4.14	32.73	153.47	4.15	41.83	42.93	0.95	11.70	366.91
W_3_N_1_	48.44	1.73	11.88	126.70	4.37	31.08	178.16	4.82	43.71	54.30	1.21	13.32	407.60
W_2_N_0_	49.52	1.77	13.29	122.90	4.24	32.99	155.60	4.21	41.77	44.49	0.99	11.94	372.51

**Table 6:** The correlation coefficients.

Index	x_1_	x_2_	x_3_	x_4_	x_5_	x_6_	x_7_	x_8_	x_9_	x_10_
**x_1_**	-									
**x_2_**	0.935 **	-								
**x_3_**	−0.942 **	−0.938 **	-							
**x_4_**	0.853 **	0.862 **	−0.845 **	-						
**x_5_**	0.932 **	0.952 **	−0.947 **	0.830 **	-					
**x_6_**	0.784 **	0.761 **	−0.770 **	0.613 **	0.797 **	-				
**x_7_**	0.784 **	0.786 **	−0.783 **	0.615 **	0.826 **	0.784 **	-			
**x_8_**	0.947 **	0.876 **	−0.908 **	0.765 **	0.877 **	0.776 **	0.803 **	-		
**x_9_**	0.896 **	0.806 **	−0.842 **	0.689 **	0.818 **	0.729 **	0.761 **	0.985 **	-	
**x_10_**	0.798 **	0.710 **	−0.808 **	0.731 **	0.760 **	0.634 **	0.664 **	0.707 **	0.608 **	-

**Note:** “**” means extremely significant (*p* ≤ 0.01).

**Table 7:** The membership function method.

Treatment	Membership Function Value									
	R(x_1_)	R(x_2_)	R(x_3_)	R(x_4_)	R(x_5_)	R(x_6_)	R(x_7_)	R(x_8_)	R(x_9_)	R(x_10_)
W_1_N_2_	0.145	0.160	0.307	0.191	0.234	0.262	0.129	0.237	0.307	0.046
W_2_N_2_	0.659	0.600	0.716	0.635	0.559	0.500	0.318	0.782	0.803	0.452
W_3_N_2_	0.884	0.931	0.962	0.808	0.971	0.857	0.624	0.865	0.836	0.629
W_1_N_1_	0.000	0.112	0.114	0.270	0.129	0.071	0.021	0.000	0.000	0.147
W_2_N_1_	0.420	0.439	0.640	0.511	0.454	0.429	0.253	0.445	0.390	0.618
W_3_N_1_	0.710	0.616	0.826	0.692	0.739	0.548	0.422	0.642	0.581	0.943
W_2_N_0_	0.333	0.338	0.438	0.453	0.397	0.405	0.228	0.314	0.337	-

**Table 8:** Principal component analysis.

Component	Eigenvalue	Variance (%)	Contribution Rate (%)
1	8.399	83.989	83.989
2	0.574	5.744	89.733
3	0.387	3.871	93.604
4	0.242	2.419	96.023
5	0.204	2.035	98.059
6	0.113	1.134	99.193
7	0.047	0.466	99.659
8	0.027	0.268	99.927
9	0.006	0.062	99.989
10	0.001	0.011	100.000

**Table 9:** Principal component matrix.

Index	Principal Component	
	1	2
x_1_	0.987	−0.038
x_2_	0.953	−0.078
x_3_	−0.967	0.109
x_4_	0.863	−0.383
x_5_	0.964	−0.046
x_6_	0.869	0.316
x_7_	0.859	0.277
x_8_	0.962	0.15
x_9_	0.915	0.258
x_10_	0.807	−0.375

**Table 10:** The comprehensive scores of different treatments.

Treatment	Principal Component Analysis		Membership Function Method	
	Comprehensive Score	Comprehensive Ranking	Comprehensive Score	Comprehensive Ranking
W_1_N_2_	−2.035	6	0.202	6
W_2_N_2_	1.486	3	0.602	3
W_3_N_2_	4.004	1	0.837	1
W_1_N_1_	−3.851	7	0.086	7
W_2_N_1_	−0.287	4	0.460	4
W_3_N_1_	1.538	2	0.672	2
W_2_N_0_	−0.856	5	0.324	5

**Table 11:** Physical and chemical soil indexes.

Soil Layer (cm)	Available *p* (mg·kg^−1^)	Available K (mg·kg^−1^)	Alkaline Hydrolysis N (mg·kg^−1^)	Organic Matter (g·kg^−1^)	Bulk Density (g·cm^−3^)
0–20	23.77	199.06	42.98	14.86	1.35
20–40	22.17	169.33	32.04	14.28	1.43
40–60	22.86	114.46	43.41	9.73	1.44

**Table 12:** Experimental design.

Treatment	Irrigation Quota (mm)					Irrigation Cycle (d)				Fertilizer-N Application Rate (kg·ha^−1^)
	Sowing–Emergence Stage	Seedling Stage	Flowering–Pegging Stage	Pod-Setting Stage	Pod-Filling Stage	Seedling Stage	Flowering–Pegging Stage	Pod-Setting Stage	Pod-Filling Stage	
W_1_N_2_	45	-	22.5	22.5	22.5	-	10	10	15	110
W_2_N_2_	45	-	30	30	30	-	10	10	15	110
W_3_N_2_	45	-	37.5	37.5	37.5	-	10	10	15	110
W_1_N_1_	45	-	22.5	22.5	22.5	-	10	10	15	77.5
W_2_N_1_	45	-	30	30	30	-	10	10	15	77.5
W_3_N_1_	45	-	37.5	37.5	37.5	-	10	10	15	77.5
W_2_N_0_	45	-	30	30	30	-	10	10	15	0

**Table 13:** The actual irrigation and fertilization times, irrigation volumes, and nitrogen application rates during the peanut growth period.

Day after Sowing (d)	Irrigation Quota (mm)							Fertilizer–N Application Rate (kg·ha^−1^)						
	W_1_N_2_	W_2_N_2_	W_3_N_2_	W_1_N_1_	W_2_N_1_	W_3_N_1_	W_2_N_0_	W_1_N_2_	W_2_N_2_	W_3_N_2_	W_1_N_1_	W_2_N_1_	W_3_N_1_	W_2_N_0_
1	45.00	45.00	45.00	45.00	45.00	45.00	45.00	45	45	45	45	45	45	-
32	25.34	30.93	23.02	24.91	27.53	28.36	19.57	16.25	16.25	16.25	8.125	8.125	8.125	-
42	23.27	30.00	42.28	22.90	31.11	37.50	30.00	16.25	16.25	16.25	8.125	8.125	8.125	-
53	22.50	30.00	37.50	22.50	30.00	37.50	30.00	16.25	16.25	16.25	8.125	8.125	8.125	-
62	22.50	30.00	37.50	22.02	30.00	37.50	30.00	16.25	16.25	16.25	8.125	8.125	8.125	-
74	22.50	33.15	37.50	22.50	30.00	38.67	30.00	-	-	-	-	-	-	-
84	22.50	37.53	46.90	24.00	37.53	45.09	37.53	-	-	-	-	-	-	-
101	30.00	37.53	45.00	30.49	37.53	46.57	39.26	-	-	-	-	-	-	-
118	22.50	30.00	37.50	23.49	30.00	37.99	30.00	-	-	-	-	-	-	-
130	22.50	22.53	24.50	22.53	30.53	22.53	22.53	-	-	-	-	-	-	-

## Data Availability

The data are contained within the article.
